# 3D-printed navigation template in proximal femoral osteotomy for older children with developmental dysplasia of the hip

**DOI:** 10.1038/srep44993

**Published:** 2017-03-21

**Authors:** Pengfei Zheng, Peng Xu, Qingqiang Yao, Kai Tang, Yue Lou

**Affiliations:** 1 Department of Pediatric Orthopaedics, Children’s Hospital of Nanjing Medical University, Nanjing, Jiangsu, 210008, China; 2 Digital Medicine Institute, Nanjing Medical University, Nanjing, Jiangsu, 210006, China; 3 Department of Orthopedics, Nanjing First Hospital, Nanjing Medical University, Nanjing, Jiangsu, 210006, China

## Abstract

To explore the feasibility of 3D-printed navigation template in proximal femoral varus rotation and shortening osteotomy for older children with developmental dysplasia of the hip (DDH). Between June 2014 and May 2015, navigation templates were designed and used for 12 DDH patients. Surgical information and outcomes were compared to 13 patients undergoing the same surgery but without navigation template. In template-guided patient group, operation time (21.08 min vs. 46.92 min), number of X-ray exposures (3.92 vs. 6.69), and occurrence of femoral epiphysis damage (0 vs. 0.92) were significantly decreased (P < 0.05). Furthermore, after 12–18 months follow-up, 66.7% and 16.7% of the hips in template-guided group were rated as excellent or good, respectively, according to the McKay criteria; 83.3% and 16.7% by using the Severin criteria respectively. By contrast, 46.2% and 23.1% of the hips in traditional operation group were classed as excellent or good, respectively, using the McKay criteria; 46.2% and 30.8% by using the Severin criteria respectively. The template-guided group achieved a better outcome; however, there was no significant difference. Application of the navigation template for older DDH children can reduce the operation time, radiation exposure, and epiphysis damage, which also simplifies surgery and improves precision.

Developmental dysplasia of the hip (DDH), the most common hip malformation in children, has a great number of possible complications, especially for patients who are more than eight years old when they are diagnosed and treated because of the special anatomic structure of DDH^1^,^2^. Periacetabular osteotomy combined with proximal femoral varus rotation and shortening osteotomy is a commonly used surgical technique for older DDH patients; however, the typically narrow, deformed femoral neck and the high level of variability between individuals pose surgical challenges. The success and precision of proximal femoral varus rotation and shortening osteotomy is mainly based on the experience of orthopedic surgeons. This entails both excellent preoperative planning and intraoperative skills. Any error of preoperative planning or intraoperative manipulation will affect surgical outcomes. For these reasons, a more precise but simplified surgical method is needed.

Rapid prototyping (RP) technology is an emerging industrial technology. Its application in combination with reverse engineering technology in the field of medicine makes precise, individualized treatment possible. This is especially important for children, who show great individual physical differences. Preoperative parameter measurement and surgical planning, along with the application of a 3D-printed navigation template, can effectively reduce errors associated with inexperience and poor operative technique. Currently, navigation templates are generally used to guide the surgical insertion of internal fixation screws and plates^3^,^4^,^5^; however, its application for proximal femoral varus rotation and shortening osteotomy in older DDH patients has not been reported. The aim of this study was to use digital 3D reconstruction and reverse modeling technology combined with 3D printing to produce a navigation template for this purpose. The template was designed to conveniently include all the necessary parameters and procedural steps to serve as a guide for the operation.

## Materials and Methods

### General Information

This research was authorized by Nanjing Children’s Hospital’s ethics committee, and all patients and their parents/legal guardians were given detailed information about the study and their situation. All the parents/legal guardians signed the informed consent forms. The 3D navigation template was used as part of the surgical procedure for 12 older DDH patients (12 hips; 8 left, 4 right; 2 males, 10 females) (template-guided group). The average patient age was 10.9 years (range, 8–14 years). The degree of hip dislocation was determined using the Tönnis classification system: 2 hips were classed as grade II; 6 hips were grade III; and 4 hips were grade IV. Six of the cases were primary surgeries, 4 cases were performed after the failure of conservative treatment and 2 were performed due to the recurrence of hip dysplasia after Salter pelvic osteotomy. Another 13 older DDH patients that were undergoing the same surgery at the same time as the template-guided group but without the aid of navigation templates were also studied (traditional operation group). There was no significant difference between the two groups in terms of age, gender, side, Tönnis classification, and surgery history (Table 1).

### Preoperative Measurement, Design and Template Preparation

**Figure 1:**
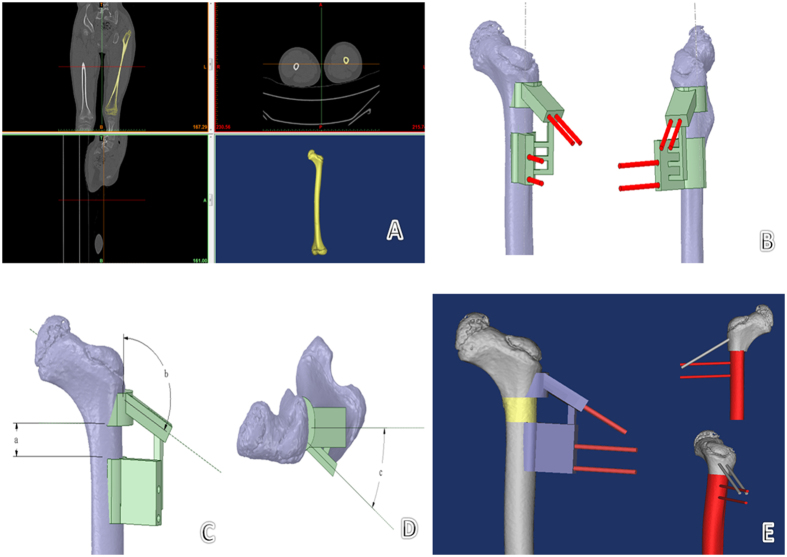
Preoperative design and template preparation. (**A**) DICOM data were imported into Mimics software for 3D reconstruction. (**B**) The bone cutting plane and needle insertion channel were simulated; the navigation template was designed with reverse modeling. A Boolean operation was used to acquire femoral surface morphology, and the 3D navigation template model was set up with the insertion channel for the Kirschner wire. (**C,D**) The navigation template includes all the operation parameters and steps for proximal femoral varus rotation and shortening osteotomy: (a) is the shortening length of bone cutting, (b) is the needle insertion angle on the femoral neck (b = LCP-PHP angle + varus angle), and (c) is the rotation angle of bone cutting. (**E**) The osteotomy process was simulated using the navigation template in Mimics software using the Kirschner wire as a lever.

All experimental methods described in this study were approved by Nanjing Children’s Hospital and were performed in accordance with the relevant guidelines and regulations. The preoperative parameters—actual femoral length, bilateral acetabulum index, and height of the femoral head dislocation—were measured on pelvic X-ray. A spiral CT scan of the pelvis and femur was obtained using specific scan parameters (120 kV; 120 mAs; pixel matrix, 512 × 512; 1-m-thick slices, 0.5 mm interlamellar spacing). Original DICOM data were imported into Mimics 17.0 software (Materialise, Belgium) to produce a 3D reconstruction of the femur. A simulation module was used to measure both the angles of femoral anteversion and the neck-shaft. These data were imported into the 3D reconstruction program (STL format) in Geomagic Design Direct software to redefine the coordinate axis. According to the preoperative measurements and in comparison with the contralateral parameters, the femoral varus angle, rotation angle, and length of bone to be cut were determined. A Boolean operation was used to acquire the femoral surface morphology. The simulated bone cutting plane and channel for needle insertion refer to the parameters of the Locking Compression Pediatric Hip Plate (LCP-PHP; Synthes, Switzerland), which will be used for fixation. A navigation template with reverse modeling was designed, and the navigation template 3D model was completed with the insertion channel for Kirschner wire needles (Fig. 1). The template and femur model were produced using the fused deposition molding (FDM) method of RP technology.

### Simulated Operation

**Figure 2:**
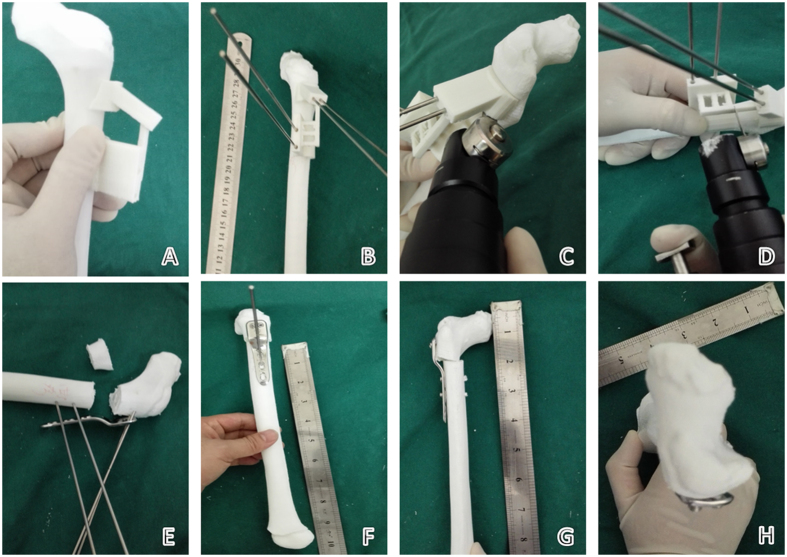
Simulated operation with the 3D-printed model and navigation template. (**A**) The navigation template was matched to verify the degree of surface feature matching. (**B**) The Kirschner wire needle was placed according to the navigation hole. (**C,D**) The bridge part of the navigation template and femoral model was removed according to the bone cutting plane in the navigation template, and the cutting section and navigation template were removed. (**E**) The Kirschner wire needle was used as a lever through the LCP-PHP corresponding screw hole to complete the varus rotating and shortening osteotomy. (**F**) The positioning needles were removed one by one using the Kirschner wire needle pinhole as the screw nail hole, screwing the screw to the preoperative predicted length and completing internal fixation. (**G,H**) After the simulation was complete, postoperative angles of femoral anteversion and neck-shaft and shortening length were consistent with the preoperative plans.

After matching navigation templates to the models of the proximal femur, Kirschner wire needles were inserted through the navigation holes. The bridge portions of the navigation template and femoral model were cut along the guide on the template. The cut sections and the navigation template were removed. The previously inserted wire needles were used as a lever to achieve proper positioning and orientation according to the preoperative measurements. The positioning needles were removed one at a time and, using the Kirschner wire needle pinhole as a screw hole, screws of a preoperatively determined length were inserted and tightened to complete the internal fixation with the LCP-PHP (Fig. 2).

### Operation and Postoperative Treatment

**Figure 3:**
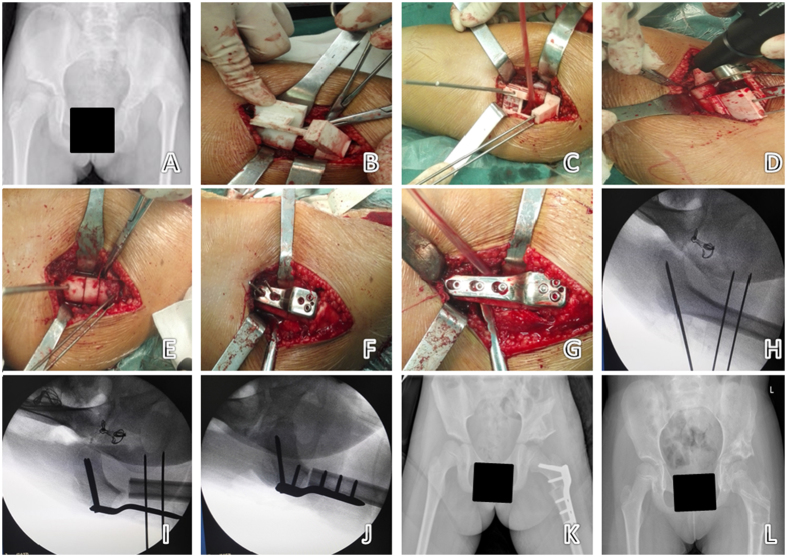
The navigation template applied in the operation on a 9-year-old girl with left DDH. (**A**) Preoperative X-ray of the pelvis. (**B–G**) According to the simulation steps, the steps of the intraoperative process were completed as performed in the simulated operation. (**H–J**) Intraoperative use of C-arm X-ray to verify the direction of the needle and bone cutting form, consistent with preoperative planning (only 3 X-ray exposures). (**K**) X-ray of the pelvis one week after surgery. (**L**) X-ray of the pelvis 14 months after surgery.

Using a lateral femoral longitudinal incision, the lateral muscle was cut and carefully separated to reveal the base of greater trochanter where has a “step” with femoral shaft. The 3D navigation template was used to perform the varus rotation and shortening osteotomy as described above (see *Simulated Surgery*), and an LCP-PHP was aligned with the wire needles. The needles were removed in turn, and the resected femur was fixed with screws (Fig. 3).

Postoperative management procedures were the same in the two groups of patients after Pemberton acetabulum osteotomy was completed. A long double lower limb brace with hip abduction was used. Progressive hip flexion function exercises were begun after 2 weeks. Postoperative removal of the brace was performed after 8 weeks, and weight bearing was initiated after 12 weeks. After a minimum of six months, when the osteotomy had completely healed, the internal fixation material was removed.

### Statistical Analysis

All measurement data were presented as the mean ± the SD and Student’s t-test was used to examine the differences between groups. All count data between the two groups were compared using the chi square test or Fisher’s exact test analysis. All statistical analyses were carried out using Stata software, version 9.0 (StataCorp LP, College Station, TX, USA) and SPSS software, version 17.0 (SPSS Inc., USA). Statistical significance was set at p < 0.05.

## Results

### Preoperative Measurement and Simulation Operation

Using 3D CT image reconstruction, precise measurements of femoral parameters were obtained. The patients’ average femoral neck anteversion angle was 53.33° (range, 44°–68°) and average neck–stem angle was 145.83° (128°–160°), which were consistent with the actual intraoperative findings. The precise location of the bone cutting plane and needle insertion channel, as well as the necessary needle depth into the bone, were all successfully calculated using the computer. Furthermore, the 3D printer accurately produced the femur model and navigation template using medical PLA (polylactic acid) material. The needle channels in the template were perfectly matched with the screw holes of the LCP-PHP and varus, and rotating, shortening osteotomy, and internal fixation were easily completed. The observation of the femur model showed that the femoral neck anteversion angle and neck–stem angle were returned to normal and were consistent with the computer design (Fig. 2).

### Proximal Femoral Varus Rotation and Shortening Osteotomy

In the template-guided patient group, the average femoral varus was 14.16° (range, 10°–20°); the average rotation was 27.50° (range, 25°–45°); and the average shortening length of the bone was 2.21 cm (range, 1.5–2.5 cm). The average operation time (from exposure of the greater trochanter until the cortical screw was tightened in place) was 21.08 min (range, 14–32 min). Intraoperative X-ray exposure occurred 3.92 times (range, 3–5 times), and there was no femoral epiphysis damage. In the traditional operation group of patients, the average femoral varus was 14.23° (range, 10°–20°); the average rotation was 32.69° (range, 25°–45°); and the average shortening of the bone was 2.21 cm (range, 1.5–3.0 cm). The average operation time (from the exposure of the greater trochanter until the cortical screw was tightened in place) was 46.92 min (range, 32–70 min). Intraoperative X-ray exposure occurred 6.69 times (range, 5–10 times) and femoral epiphysis damage occurred 0.92 times (range, 0–3 times). There were significant decreases in operation time (P < 0.001), X-ray exposure times (P < 0.001) and the occurrence of femoral epiphysis damage (P = 0.008) in template-guided patient group when compared with traditional operation group patients (Table 2).

### Surgical Outcome

Surgical outcome was assessed 12 to 18 months following surgery using the McKay clinical classification system^6^ and the Severin radiographic scale^7^. In the template-guided group, the hips of 8 patients were rated as excellent (66.7%), 2 as good (16.7%), and 2 as fair (16.7%) using the McKay classification system. Using the Severin scale, the hips of 10 patients were scored as excellent (83.3%) and two as good (16.7%). There were no significant osteotomy-related complications, such as redislocation or avascular necrosis. In the traditional operation group, the hips of 6 patients were rated as excellent (46.2%), 3 as good (23.1%), 2 as fair (15.4%), and 2 as poor (15.4%) using the McKay classification system. Using the Severin scale, the hips of 6 patients were scored as excellent (46.2%), 4 as good (30.8%), 2 as fair (15.4%), and 1 as poor (7.6%). One case suffered the complication of subdislocation in the traditional operation group. The template-guided group achieved a better surgical outcome; however, there was no significant difference in surgical outcome between the two groups (McKay, P = 0.484; Severin, P = 0.201; Table 2).

## Discussion

From a technical standpoint, the treatment for DDH is the concentric reduction of the acetabular and femoral head with the goal of restoring normal anatomy to allow full function of the hip joint. With this disorder, the potential for normal development of the hip gradually decreases with age because, by eight years of age, the acetabulum is no longer capable of reshaping^8^. Therefore, older children (>8 years) with DDH have higher rates of severe complications, which may include joint stiffness, ischemic necrosis of the femoral head, and redislocation. Proximal femoral varus rotation and shortening osteotomy is used to correct the larger neck-shaft angle and anteversion angle, and high femoral dislocation is found in older children with DDH. It is essential that each step in the process is accurate to achieve the goal of concentric reduction of the acetabular and femoral head. Furthermore, the exact placement of the LCP-PHP after osteotomy is difficult, often with long operation times and high levels of X-ray exposure needed to achieve the best result. Therefore, the outcome of traditional surgery has been dependent on the experience and skill of the surgeon. Lack of accuracy may prolong the operation or lead to additional operations, which can affect the proximal femoral blood supply and the stability of the internal fixation, as well as the surgical efficacy. Therefore, there has been an urgent need for a new surgical method, such as a navigation template, to improve the precision and speed of the procedure.

With the development of digital medical and 3D printing technologies, orthopedic medicine is moving towards the use of personalized, accurate, minimally invasive technologies^9^. Reverse modeling and RP technologies are widely used both at home and abroad to produce 3D-printed surgical navigation templates^10^,^11^,^12^,^13^. However, there have been no reports of their use in the treatment of older children with DDH.

Our research group has successfully prepared a navigation template for screw placement to be used in the treatment of pediatric femoral neck fracture^14^. It allows for accurate one-time intraoperative internal fixation. Shi Qiang *et al*.^15^ used CT data to design the best osteotomy plane for femoral rotation osteotomy, to design a navigation template for the proximal femoral rotation osteotomy, intraoperatively correcting the femur anteversion angle. However, it has not been combined with the size of the varus, shortening osteotomy, and internal fixation plate.

Based on this research, our research group added two osteotomy planes to the navigation template to achieve accurate shortening. In addition, accurate varus osteotomy was achieved by the precise angle of the screws through the femoral neck, which was calculated according to the difference in the bilateral neck-shaft angle and the LCP-PHP type. The intraoperative use of a needle as a lever to complete varus and rotation helps to reduce the number of operative steps. The distance between each hole for guided needles on the navigation template was calculated in combination with the parameters of the LCP-PHP, which will be used for the operation. Guided wire channels were used as entry points for screws to complete the LCP-PHP fixation after varus rotation and shortening osteotomy in one step, greatly reducing the operation time and intraoperative X-ray exposure to the medical staff and children. Because the distance between the femoral epiphysis and entry point had been calculated before the operation, the lengths of Kirschner wire needles or screws were controlled during the operation so that no femoral epiphysis damage occurred. The postoperative femoral proximal varus, rotation angle, and shortening of the bone were consistent with the preoperative computer design in this group of 12 patients, and this high precision assured the effectiveness of the operation. Short-term follow-up results using the McKay and Severin standards were better than the traditional operation group; however, there was no significant difference between the groups. This may be because: first, the numbers of patients in each group were too small to establish significance. When we compared only the ratio of excellent outcomes between the two groups, the ratios were 66.7% vs. 46.2% using the McKay criteria and 83.3% vs. 46.2% using the Severin criteria. Future studies should include larger sample sizes. Second, the follow-up time was too short. Some complications, such as coxarthrosis, which mostly occurs as a consequence of acetabular dysplasia, would be extremely worrying in the future because most of these patients cannot be treated by total hip arthroplasty before they are 50 years old^16^. Therefore, a longer duration of follow-up is needed. Third, in addressing osteotomy in older children with DDH, acetabular osteotomy has the same effect on prognosis as femoral osteotomy. Therefore, our future research should focus on accurate acetabular osteotomy using Computer Aided Design (CAD) and 3D printing technology to realize true concentric reduction.

Children are different from adults, and different ages have specific anatomical features with individual variations. Furthermore, growth imbalances or angular deformity will appear if the epiphysis is damaged during the operation. Computer-assisted image reconstruction technology with reverse modeling 3D printing technologies will help surgeons address the individual characteristics and pathological changes among children and can simplify procedures and improve operation accuracy by preparing appropriate surgical navigation templates. There are several advantages of using the navigation template designed for this study for proximal femoral varus rotation and shortening: (1) the navigation template specifies the necessary parameters and operative steps, which should help improve surgical precision and efficacy. (2) The needles used in the procedure are later used as a lever, and the needle channel also serves as a path for the placement of screws for LCP-PHP fixation. These factors shorten the operation time, decrease intraoperative X-ray exposure and decrease surgical risk. (3) During the procedure, the surgeon follows the stepwise guide of the navigation template to complete the operation. This reduces the negative effect of inexperience (e.g., in a young doctor) on outcome. This advantage particularly increases its clinical value in pediatric orthopedics.

## Additional Information

**How to cite this article**: Zheng, P. *et al*. 3D-printed navigation template in proximal femoral osteotomy for older children with developmental dysplasia of the hip. *Sci. Rep.* **7**, 44993; doi: 10.1038/srep44993 (2017).

**Publisher's note:** Springer Nature remains neutral with regard to jurisdictional claims in published maps and institutional affiliations.

## Figures and Tables

**Table 1:** Comparison of general information relating to the template-guided group and traditional operation group.

	Age	Gender		Side		Tönnis classification				Primary surgery	
	(years)	M	F	L	R	I	II	III	IV	Yes	No
Template-guided group (n = 12)	10.85 ± 2.02	2	10	8	4	0	2	6	4	6	6
Traditional operation group (n = 13)	10.48 ± 1.99	2	11	10	3	0	2	8	3	8	5
p value	0.646	1.0		0.673		0.823				0.561	

(M = male; F = female; L = left; R = right).

**Table 2:** Comparison of operation information and results of the template-guided group and traditional operation group.

	Varus angle	Rotation angle	Shortening length	Operation time	X-ray exposure	Epiphyseal injury	McKay standard				Severin standard			
	(°)	(°)	(cm)	(min)	(times)	(times)	E	G	F	P	E	G	F	P
Template-guided group (n = 12)	14.16 ± 2.89	27.50 ± 6.57	2.21 ± 0.32	21.08 ± 4.64	3.92 ± 0.90	0	8	2	2	0	10	2	0	0
Traditional operation group (n = 13)	14.23 ± 4.00	32.69 ± 6.65	2.21 ± 0.47	46.92 ± 11.51	6.69 ± 1.49	0.92 ± 1.12	6	3	2	2	6	4	2	1
p value	0.964	0.062	0.997	<0.001	<0.001	0.008	0.484				0.201			

(Operation time = from exposure of the greater trochanter until the LCP-PHP was fixed. E = excellent; G = good; F = fair; P = poor).
